# A closer look at heterotopic sites of pleomorphic adenoma, particularly in women: fide, sed cui vide?

**DOI:** 10.1590/1806-9282.20231193

**Published:** 2024-03-04

**Authors:** Ilker Sengul, Demet Sengul, José Maria Soares

**Affiliations:** 1 Giresun University, Faculty of Medicine, Division of Endocrine Surgery – Giresun, Turkey.; 2 Giresun University, Faculty of Medicine, Department of General Surgery – Giresun, Turkey.; 3 Giresun University, Faculty of Medicine, Department of Pathology – Giresun, Turkey.; 4 Universidade de São Paulo, Faculdade de Medicina, Hospital das Clínicas, Departamento de Obstetrícia e Ginecologia, Disciplina de Ginecologia, Laboratório de Ginecologia Estrutural e Molecular – São Paulo (SP), Brazil.

The majority of the minor salivary gland tumors are malignant but they are not frequent in overall salivary gland tumors. We read with a great deal the article entitled “Heterotopic sites of pleomorphic adenoma.” This beneficial report on two vignette cases of women determined heterotopic sites and exhibited two locations, i.e., the level IIA of the second compartment of the neck and the lower lip. Kaur and colleagues declared in their recent work that the provisional diagnosis for a 46-year-old woman with lower lip swelling performed by the clinician was a mucocele, *a priori*. Afterward, the authors stated that fine-needle aspiration (FNA) cytology was applied, and a diagnosis of pleomorphic adenoma was inferred. Finally, they reported that the patient was lost to follow-up, *a posteriori* ^1^. Kaur et al. also emphasized that pleomorphic adenoma, *per se*, is rarely seen in males; the lip is a rare site of occurrence for this phenomenon; the lower lip is a more seldom location than the upper one; the upper lip tumors are more commonly benign, whereas the lower lip ones are more frequently malignant due to their difference in embryonic development; and the complete wide local resection of the tumor with negative margins is the treatment of choice^1-4^. They remarked in their work that they opted for an FNA procedure and lost her to follow-up as she possessed a benign cytology. Nevertheless, age over 40 years, male sex, mass over 20 mm in diameter, locations of the deep lobe, and inefficient first surgical intervention might be the influencing factors of multiple recurrences in pleomorphic adenoma^1-5^. In addition, possible malignant transformation is estimated at the rate of 2–7%. Of note, deficiency of encapsulation might lead to a rupture and seeding of the mentioned tumoral structure^2-4^. Herewith, we postulate that at least a meticulous and long-term follow-up is crucial for not overlooking any recurrence or malignant transformation, particularly on the topographic localization at the lower lip, by choosing a non-surgical approach, which might bring blurred lines^6^ in the minds of providers. As a matter of fact, this issue merits further investigation. We thank Kaur et al.^1^ for their valuable study.
